# Bioimpedance Vector Analysis in Diagnosing Severe and Non-Severe Dengue Patients

**DOI:** 10.3390/s16060911

**Published:** 2016-06-18

**Authors:** Sami F. Khalil, Mas S. Mohktar, Fatimah Ibrahim

**Affiliations:** 1 Department of Biomedical Engineering, Faculty of Engineering, University of Malaya, 50603 Kuala Lumpur, Malaysia; samifathi@siswa.um.edu.my (S.F.K.); mas_dayana@um.edu.my (M.S.M.); 2 Centre for Innovation in Medical Engineering (CIME), Faculty of Engineering, University of Malaya, 50603 Kuala Lumpur, Malaysia; 3 Department of Biomedical Engineering, College of Engineering, Sudan University of Science and Technology, 407 Khartoum, Sudan

**Keywords:** bioimpedance analysis, body composition, bioimpedance vector analysis, Dengue infection

## Abstract

Real-time monitoring and precise diagnosis of the severity of Dengue infection is needed for better decisions in disease management. The aim of this study is to use the Bioimpedance Vector Analysis (BIVA) method to differentiate between healthy subjects and severe and non-severe Dengue-infected patients. Bioimpedance was measured using a 50 KHz single-frequency bioimpedance analyzer. Data from 299 healthy subjects (124 males and 175 females) and 205 serologically confirmed Dengue patients (123 males and 82 females) were analyzed in this study. The obtained results show that the BIVA method was able to assess and classify the body fluid and cell mass condition between the healthy subjects and the Dengue-infected patients. The bioimpedance mean vectors (95% confidence ellipse) for healthy subjects, severe and non-severe Dengue-infected patients were illustrated. The vector is significantly shortened from healthy subjects to Dengue patients; for both genders the *p*-value is less than 0.0001. The mean vector of severe Dengue patients is significantly shortened compare to non-severe patients with a *p*-value of 0.0037 and 0.0023 for males and females, respectively. This study confirms that the BIVA method is a valid method in differentiating the healthy, severe and non-severe Dengue-infected subjects. All tests performed had a significance level with a *p*-value less than 0.05.

## 1. Introduction

Dengue is a common arthropod-borne infection in tropical regions of the world, with 500,000 cases and approximately 12,000 deaths annually [1]. Real-time monitoring and precise diagnosis of the severity of Dengue infection is needed for better decisions in disease management. Many methods have been launched and recommended by the World Health Organization (WHO) in order to manage and cure Dengue infection [1]. Most of these methods are based on blood and laboratory tests to assess the Dengue infection status. Despite the fact that these approaches can precisely diagnose Dengue infection, most of them are expensive, invasive and time-consuming in giving results [2,3,4].

Bioimpedance analysis (BIA) is a method that is applied noninvasively, the machine is portable and it is a cost-effective technique to estimate the body composition and hydration status in the human body [5,6,7]. The BIA method utilizes different sets of multiple regression prediction equations to assess the body composition compartments [8,9]. The estimated body composition measurements from these equations may biased based on weight, age, and body anthropometric measurements [10,11]. In addition, studies stated that these prediction equations cause assessment errors in diseased subjects with alternation in body composition and hydration condition [12,13].

**Figure 1:**
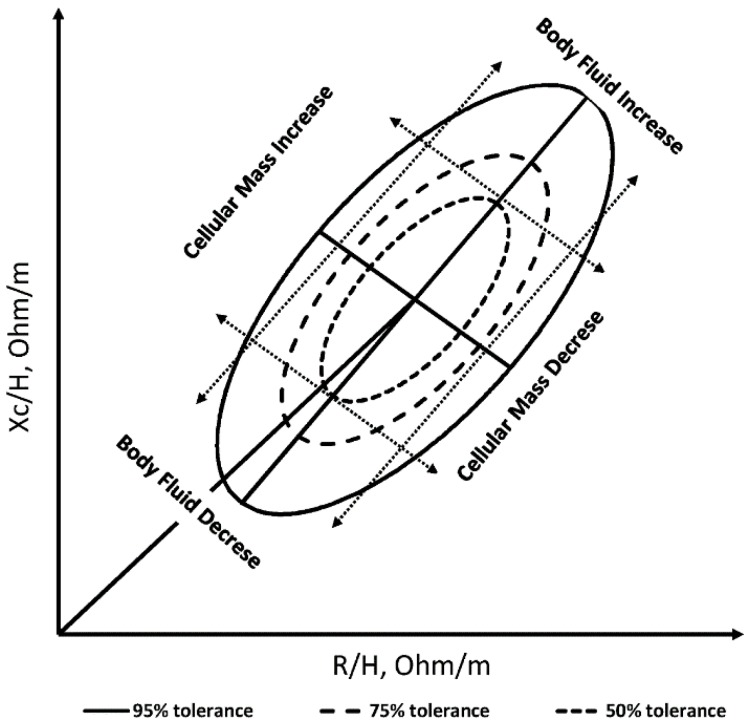
The (R-Xc) graph and tolerance ellipses. Reproduced with permission, Piccoli *et al.* [17].

Bioimpedance vector analysis (BIVA) is an independent and rapid technique to monitor the changes in body composition and hydration status in the human body. This new technique was introduced by Piccoli *et al.* [14,15]. It utilizes only the height-indexed resistance (R) and reactance (Xc) measurements to assess the body fluid and cellular mass conditions. The distribution of these measurements is plotted in an R-Xc bivariate graph for each gender separately. The gender-specific 50%, 75%, and 95% tolerance ellipses allow the analysis of the vector length and the angle. The major and minor ellipse axis reflects the alternation in cellular mass and body fluids, respectively. Separate 95% ellipses indicate different cellular mass and fluidic conditions for groups in each ellipse. The accumulation of BIVA parameter distribution on each of the ellipse quarters indicates the common characteristics of certain populations. The obese population is characterized by have high cellular mass and low body fluid compared to the lean population who have low cellular mass and high body fluid. The cachectic population is characterized by low cellular mass and body fluid compared to the athletic population with high cellular mass and body fluid [16]. Figure 1 illustrates the (R-Xc) graph and tolerance ellipses.

The BIVA method has been used in many studies, and was proofed to be valid in estimating the hydration status in healthy and diseased subjects [18,19,20,21]. Recently, Buffa *et al.* [18] reviewed 30 steady studies that utilized the BIVA method as a valid approach in assessing body composition in healthy subjects and patients. Even though some studies have utilized the BIA method in analyzing and classifying the risk in Dengue-infected patients [2,22], to date no known study utilized the BIVA analysis in the diagnosis of severe and non-severe Dengue patients. This study aims to introduce the BIVA method as a new approach for clinical monitoring of severe and non-severe hospitalized Dengue patients.

## 2. Materials and Methods

### 2.1. Subjects

In this study, data from two groups of Malaysian subjects were used; the first group is the healthy control, and the second is the Dengue-infected group. The first group consists of 299 volunteer healthy subjects (124 males and 175 females) without history of illness [23] (ethical approval number: 20147-398, University Malaya Medical Center). The second data set consists of 205 (123 males and 82 females) serologically confirmed Dengue patients on the day of defervescence, during their therapy at University Kebangsaan Malaysia (UKM) Hospital between the years 2001 and 2002 [2] (Ethics approval number: D-004-2002, University Kebangsaan Malaysia Hospital). The second group’s data was classified into two subgroups, severe and non-severe, based on Dengue severity markers. The severity classification was performed based on the WHO guidelines for Dengue diagnosis and control [1,2]. 

### 2.2. Measurements

A BIODYNAMICS (BIA 450^®^) commercially available bioimpedance analyzer was used to measure the bioimpedance parameters (resistance and reactance). Robust, commercially available height and weight scales (accuracy of 0.1 kg and 0.5 cm) were used in this study. The Body Mass Index (BMI) for subjects was obtained by dividing the weight by the squared height measurement. 

The bioimpedance analyzer (BIA 450^®^) was calibrated before the measurement, using the impedance box supplied by the manufacturer [24]. The bioimpedance measurement was performed and the subjects were in the supine position, using Ag/Cl electrodes [24]. The current and voltage electrodes were positioned distal and proximal to the right site, respectively [25,26]. The distance between the current and voltage electrodes was kept to 5 cm of blank skin [27]. All subjects abstained from wearing any metallic items and lay on a non-conducting bed sheet. All participants were held back from food intake for 4 h and exercise for 12 h before bioimpedance measurements [24]. The mean of three measurements was taken for further processing. All other safety procedures and precautions were made and were known to all participants.

The bioimpedance measurements from the Dengue-infected patients were obtained during hospitalization on the day of the defervescence (the period of reduction of fever; body temperature between 37.5 °C and 38 °C) [2].

### 2.3. Statistical Analysis

The BIVA method uses the height-indexed resistance and the reactance from the direct measurements of the bioimpedance vector to be plotted in the R/Xc graph [14]. The plots include the confidence intervals and the tolerance ellipses. 

The confidence interval of 95% of normal distributed resistance and reactance divided by height were utilized in this study. To test the significance difference between measurements in the same group, paired one-sample Hotelling’s T^2^ was used. To study the differences in age, weight, height and body mass index (BMI) between the two groups, an independent two-sample Student *t*-test was used. One-way analysis of variance (ANOVA) test was performed to assess the variance differences between the tested groups. The effect size and statistical power were tested and evaluated using a multivariate Roy's Largest Root method. A *p*-value less than 0.05 was used as significance level in this study. In addition, the Mahalanobis distance (D) was used to measure the distance between the sample points and the mean [28].

To compare the group’s means, and the subgroups, two-sample Hotelling’s T^2^ was used to statistically explore the significance mean difference. The statistically significant difference between impedance mean vectors can be pointed out as detached 95% confidence ellipses in the R-Xc plane (equal to a significant difference at a *p*-value less than 0.05). A non-overlapping confidence interval specifies a significance vector shifting between healthy and Dengue-infected subjects. Additionally, the 50%, 75% and 95% tolerance intervals were plotted for healthy males and females. All statistical analyses were completed using Microsoft Office Excel^®^ 2007, SPSS^®^ version 21.0 and BIVA software [17]. 

## 3. Results

The demographic and bioimpedance data for the 299 Malaysian healthy subjects (124 males and 175 females) and the 205 Dengue-infected patients (123 males and 82 females) are illustrated in Table 1. 

The healthy subjects are labeled as Group 1. Group 2 determined all of the 205 serologically confirmed Dengue-infected subjects.

Comparing the age between groups 1 and 2, the *p*-value of the independent two-sample Student *t*-test was found to be 0.31 and 0.39 for males and females, respectively. For males, the *p*-value was found to be 0.006, 0.49 and 0.007 comparing the weight, height and BMI between Groups 1 and 2, respectively. For females, the *p*-value was found to be 0.008, 0.09 and 0.19 comparing the weight, height and BMI between Groups 1 and 2, respectively. (A *p*-value less than 0.05 was used as the significance level).

The two-sample Hotelling’s T^2^ test proved a significant difference in mean vectors between healthy subjects and Dengue patients at a significance level of a *p*-value less than 0.0001. For both males and females, the confidence ellipses (95%) of the mean vectors for healthy and Dengue patients did not overlap. The mean vectors were significantly shortened from healthy subjects to Dengue patients for both genders. The vector end point was shifted from (289.6, 37.3) to (276.2, 32.7) in males, and from (376.1, 41.6) to (332.3, 35.4) in females.

**Figure 2:**
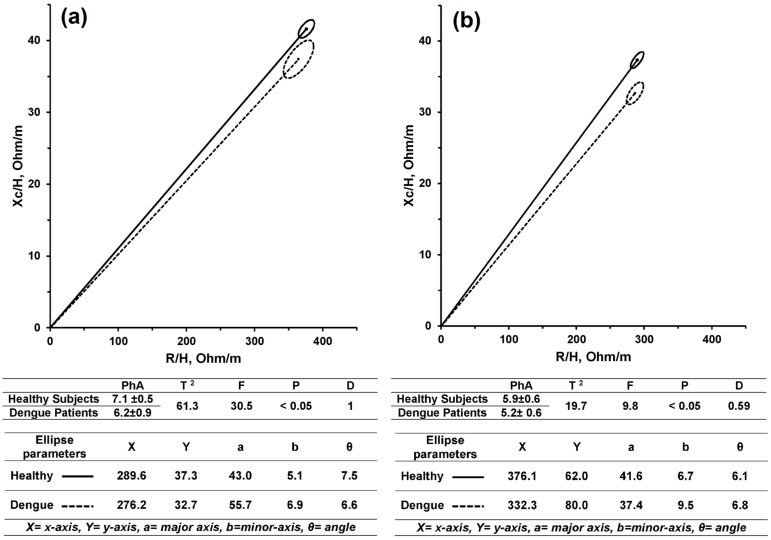
The 95% confidence ellipses for healthy (solid) and Dengue-infected (dashed) subjects: (**a**) Males; (**b**) Females.

The statistical power test shows that 83.5% of the differences between healthy subjects and Dengue-infected patients were significantly assessed by height-indexed R and Xc. The effect size was estimated to be significant and marginally moderate (η^2^ = 0.05, *p*-value = 0.005) in size when reporting the variation between healthy subjects and Dengue patients. The D-distance between healthy and Dengue infected subjects was found to be equal to 1.0 and 0.59 for males and females, respectively. Figure 2 illustrates the non-overlapping 95% confidence ellipses between healthy (solid) and Dengue-infected (dashed) subjects, for females (Figure 2a) and males (Figure 2b).

Group 2 was classified into two subgroups, severe subjects (50 males and 37 females) and non-severe subjects (73 males and 31 females). Table 2 illustrates the demographic and BIVA parameters of severe and non-severe Dengue patients. 

The two-sample Hotelling’s T^2^ test approved a significant difference in mean vectors between severe and non-severe Dengue patients at *p*-values found to be 0.0037 and 0.0023 for males and females, respectively. The statistical power using the multivariate Roy’s Largest Root test shows 97.8% of differences between severed and Non-severed Dengue patients were significantly assessed by height-indexed R and Xc. The effect size was significantly estimated to be moderate in size (η^2^ = 0.07, *p*-value less than 0.0001) when reporting the variation between severe and non-severe Dengue patients. The D-distance between non-severe and severe Dengue-infected subjects was found to be equal to 0.563 for both genders. 

**Figure 3:**
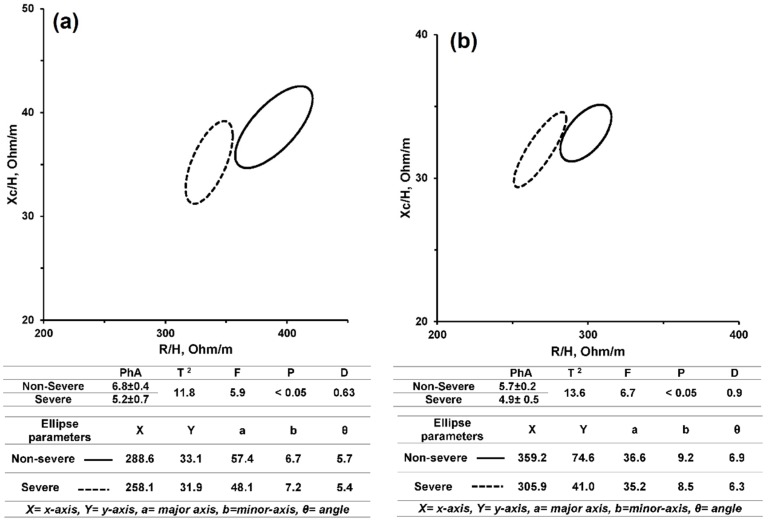
The 95% confidence ellipses for non-severe (solid) and severe Dengue-infected (dashed) subjects: (**a**) Males; (**b**) Females.

For both males and females, the confidence ellipses (95%) of mean vectors for severe and non-severe Dengue-infected patients did not overlap, which means that the mean vector coordinates of the severe and non-severe subgroups were significantly different at a *p*-value less than 0.05 using the BIVA method. Figure 3 illustrates the non-overlapping 95% confidence ellipses between non-severe (solid) and severe Dengue-infected (dashed) subjects, for females (Figure 3a) and males (Figure 3b).

**Figure 4:**
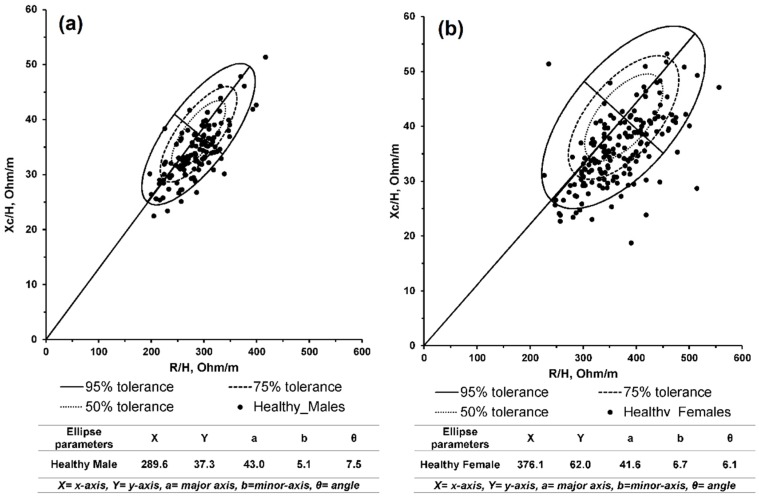
The reference ellipses (50%, 75% and 95%) for healthy subjects: (**a**) Males; (**b**) Females.

In order to compare and analyze the confidence ellipses between healthy and Dengue-infected subjects and severe and non-severe Dengue-infected subjects, the reference ellipses (50%, 75% and 95%) are presented. Figure 4 illustrates the reference ellipses for females (Figure 4a) and males (Figure 4b) with mean points of (289.6, 37.3) and (376.1, 41.6) for males and females, respectively. 

In this study, the extracellular fluid to intracellular fluid ratio (ECF/ICF) was significantly increased from healthy to severe Dengue patients at a significance level of a *p*-value less than 0.05. This increase is combined with a significant decrease in body weight for both gender groups. Table 3 shows the analysis of variance and robust Welch’s test for equality means for the weight, ECF/ICF ratio, phase angle and BIVA parameters.

## 4. Discussion

Dengue infection is a spreading disease with a wide range of intense and bland clinical indexes [29]. After the Dengue infection, the sickness passes through three stages of feverish, acute and recovery [1]. In the feverish or febrile stage, the patient develops a high-temperature fever and other related symptoms which last for two to seven days. In the acute or critical stage, the body temperature drops down and remains low, between 37.5 and 38 °C, commonly for three to seven days of sickness. At the beginning of this stage (the day of defervescence), rising hematocrit ratios combined with high capillary permeability levels will occur [30,31]. During this stage, significant plasma leakage continues for one to two days. The level of plasma leakage depends on the amount of fluid therapy and can be diagnosed using X-ray and ultrasound technologies. Because of the invasiveness, expensiveness and bulkiness of these technologies, alternative approaches are needed. Bioimpedance vector analysis utilizes the noninvasive, portable and cost-effective bioimpedance analyzer machines, and can overcome the limitations to estimating the hydration status in the human body. 

The results from this study show a significant BIVA vector shifting between the healthy and Dengue-infected subjects at a *p*-value less than 0.0001 for both males and females. The non-overlapping ellipses between severe and non-severe Dengue-infected subjects show a significant result in utilizing the BIVA method in Dengue severity classification with *p*-values of 0.0037 and 0.0023 for males and females, respectively. Furthermore, the 50%, 75% and 95% reference ellipses of bioimpedance vectors for healthy Malaysians are illustrated. 

Utilizing these reference ellipses, other abnormalities in body fluid and cell mass variations can be diagnosed. Further studies are needed to determine the reference ellipses for all age groups including children and the elderly. 

As far as we know, to date no studies using the BIVA method have been done on Dengue patients. The main contribution of this study is to prove the validity of the BIVA method in discrimination between healthy subjects and Dengue patients, and in classification between severe and non-severe Dengue subjects. 

**Figure 5:**
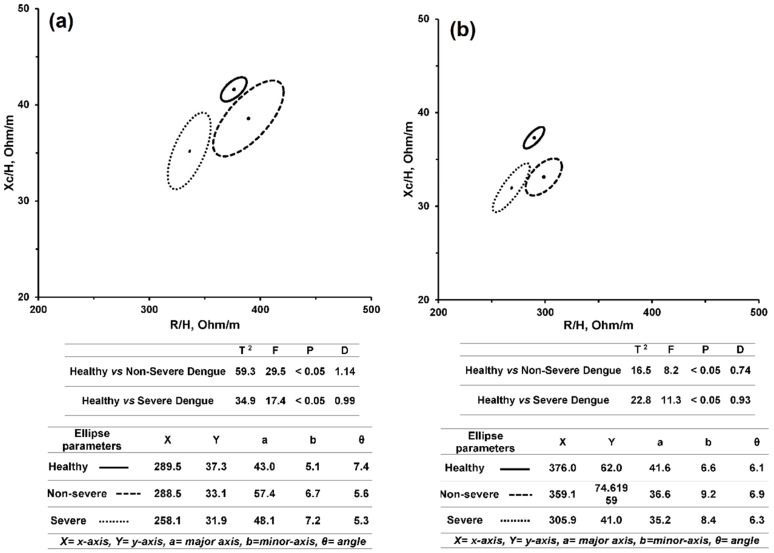
The ellipses allocation for healthy (solid), non-severe (dashed) and severe (dots) Dengue-infected subjects: (**a**) Males; (**b**) Females.

Many studies report the alternations in phase angle measurements between healthy and diseased subjects [13,32,33]. The phase angle changes were stated to decrease as a result of the fluid dropped in several health conditions [13,34]. These findings are supported based on this study, pointing out the significance in decreasing phase angle measurements from healthy subjects to Dengue-infected patients. As shown in Figure 5, the degree of difference in phase angle is higher in males compared to females, due to differences in fluid distribution for each gender [16,35]. The obtained results show the significance in increasing BIVA components (R/H, Xc/H) in females compared to male subjects.

The limitation of this study is the lack of age distribution among participants, in order to investigate the vector shifting in each age group. However, there are many studies use the BIVA method with a unit group to confirm the vector shifting due to diseases and aging [36,37,38]. 

The BIVA method has been validated on healthy subjects in many studies. Piccoli *et al.* [16] presented the bioimpedance vector distribution graphs by BMI, race, age and sex for a total of 10,222 participants from the United States (5261 male and 4961 female). Bosy-Westphal *et al.* [39] presented the bioimpedance vector for a total of 213,294 German adults and 15,605 children. Siváková *et al.* [40] provided BIVA data for a total of 1543 healthy Slovak participants (536 males and 1007 females), aged between 18 and 92 years. In this study, the reference 50%, 75% and 95% tolerance ellipses were presented for both Malaysian males and females, which can be a reference for comparison studies. Reference values of the bioimpedance vector for Malaysians are needed, and can be utilized to analyze the body composition to easily diagnose the abnormalities in fluid and cell mass. 

There are many studies using bioimpedance analysis on Dengue-infected patients. Ibrahim *et al.* [2] introduced a classification module based on body reactance to classify the risk in Dengue patients. The results from this study confirm the significant decrease in the height-indexed reactance measurements between healthy subjects and severe Dengue patients. Libraty *et al.* [41] stated that in Dengue-infected children, the ECF/ICF measured using ratio bioimpedance increase with the disease severity. As shown in Table 3, the ECF/ICF ratio was significantly increased from healthy subjects to severe Dengue patients at the significance level of a *p*-value less than 0.05. This increase is combined with a significant decrease in body weight for both gender groups.

Our study shows that the mean vector of Dengue-infected subjects is shortened compared to healthy subjects. For males, the mean vector point is (289.6, 37.3) compared to Dengue-infected males with a vector point of (276.2, 32.7). For females, the mean vector point is (376.1, 41.6) compared to Dengue-infected females with a vector point of (332.3, 35.4). 

The mean vector of severe subjects is shortened compare to non-severe Dengue subjects. For males, the mean vector point is (258.1, 32.0) compared to non-severe Dengue-infected males with a vector point of (288.5, 33.1). For females, the mean vector point is (329.2, 34.6) compared to non-severe Dengue-infected females with a vector point of (335.9, 36.2). 

This finding may be explained by the increase in body fluid and decrease in cell mass in Dengue patients. This is indicated by the decrease in R/H and Xc/H which fall in the cachectic region in the BIVA plane [15]. These results were consistent with the significant decrease in body weight from healthy subjects to severe Dengue patients. Many studies concluded that the changes in body fluid and cell mass in Dengue patients can be assessed by analyzing the reactance and resistance parameters [22,42,43]. Studies show that phase angle is a valid biomarker for the body and cellular health condition [32,33]. In these studies, a low phase angle is a common indication of low cellular mass and failure of cellular membrane permeability. Our observations for phase angle in this study approved the significant decrease from healthy subjects to severe Dengue patients. Figure 5 illustrates the ellipses allocation for healthy (solid), non-severe (dashed) and severe (dots) Dengue-infected subjects, for females (Figure 5a) and males (Figure 5b).

Up to date, no study has been done using the BIVA method in Dengue-infected subjects. To our knowledge, this study is pioneering the investigation of the comparison between healthy subjects and levels of severity among Dengue-infected Malaysian subjects. This study introduces the reference 50%, 75% and 95% tolerance ellipses for healthy Malaysians. These intervals can be utilized in epidemiological and clinical studies related to body composition among Malaysians. Moreover, this study fills the gap between the existence of referential studies among different ethnic groups [44,45]. The importance of reference ellipses that allow us to discriminate between normal and abnormal body compositions are assessed by bioimpedance measurements. It is necessary to notice that the obtained bioimpedance measurements in this study utilized the BIA 450^®^ bioimpedance analyzer from BIODYNAMICS^®^. This analyzer has been used and validated for BIVA method application in several studies [46,47]. 

Early diagnosis of Dengue infection can help in reducing the mortality rate caused by the disease. Effective management decisions at the early level of clinical care can help in assessing the Dengue infection level and deciding whether or not to send the person to the hospital. Referencing the WHO Dengue guidelines, early detection of plasma leakage during the acute stage is considered one of the main activities at this point of care level [1]. BIVA is confirmed to be a valid, rapid and easy method to assess the body fluid cell mass shift in Dengue-infected patients which allows for more effective disease management. 

## 5. Conclusions

In conclusion, the reference 50%, 75% and 95% tolerance ellipses for healthy Malaysians are presented. The vector displacements between healthy, severe and non-severe Dengue-infected patient are studied and discussed. The finding from this study confirms that BIVA is a new approach to classify severe and non-severe hospitalized Dengue patients. This will help the clinicians monitor and manage the Dengue patients. However, a longitudinal study during the hospitalization of Dengue-infected patients is needed to investigate the vector positioning change with disease development. Further studies are needed to determine the reference ellipses for all age groups including children and the elderly. 

## Figures and Tables

**Table 1:** Demographic and BIVA parameters for healthy (Group 1) and Dengue-infected subjects (Group 2).

	Study Groups	Group 1	Group 2	ANOVA Test		Student *t*-Test	
				F	*p*-Value	T	*p*-Value
***Males***							
****	**N**	124	123	-	-	-	-
**Mean ± SD**	**Age**	32.5 ± 9.2	31.3 ± 9.8	1.0	0.3	1.0	0.3
	**Weight**	70.4 ± 9.4	67.9 ± 16.8	4.3	<0.05	2.1	<0.05
	**Height**	169.1 ± 6.1	169.6 ± 7.0	0.5	0.5	−0.7	0.5
	**BMI**	24.7 ± 3.5	23.6 ± 5.5	0.4	0.5	2.4	<0.05
	**PhA**	7.1 ± 0.5	6.2 ± 0.9	97.6	<0.05	2.0	<0.05
	**R/H**	289.6 ± 43.0	276.2 ± 55.7	<0.05	0.9	-0.2	0.9
	**Xc/H**	37.3 ± 5.1	32.7 ± 6.9	3.9	0.1	9.9	<0.05
***Females***							
****	**N**	175	82	-	-	-	-
**Mean ± SD**	**Age**	35.4 ± 10.6	34.8 ± 11.9	4.7	0.4	2.8	0.4
	**Weight**	65.8 ± 12.5	60.2 ± 14.2	0.7	<0.05	0.8	<0.05
	**Height**	157.3 ± 5.4	158.6 ± 6.1	2.6	0.1	−1.6	0.1
	**BMI**	24.9 ± 4.9	24.0 ± 5.9	1.3	0.2	1.1	0.2
	**PhA**	5.9 ± 0.6	5.2 ± 0.6	67.4	<0.05	8.2	<0.05
	**R/H**	376.1 ± 62.5	332.3 ± 61.0	0.4	0.5	0.6	0.5
	**Xc/H**	41.6 ± 6.7	35.4 ± 8.9	3.7	<0.05	9.8	<0.05

**Table 2:** Demographic and BIVA parameters for severe and non-severe Dengue subjects. (*p*-value less than 0.05).

	Dengue Groups	Non-Severe	Severe	ANOVA Test		Student *t* Test	
				F	*p*-Value	T	*p*-Value
***Males***							
****	**N**	73	50	-	-	-	-
**Mean ± SD**	**Age**	30.2 ± 9.8	32.9 ± 9.7	2.3	0.1	1.0	0.3
	**Weight**	65.0 ± 14.8	60.0 ± 15.0	3.5	0.1	2.1	<0.05
	**Height**	169.4 ± 6.7	169.9 ± 7.3	0.2	0.7	−0.7	0.5
	**BMI**	22.7 ± 5.2	21.2 ± 4.9	2.7	0.1	2.4	<0.05
	**PhA**	6.8 ± 0.4	5.2 ± 0.7	259.4	<0.05	2.0	0.05
	**R/H**	288.6 ± 57.4	258.1 ± 48.1	9.5	<0.05	-0.2	0.9
	**Xc/H**	33.1 ± 6.7	32.0 ± 7.2	0.8	0.4	9.9	<0.05
***Females***							
****	**N**	37	31	-	-	-	-
**Mean ± SD**	**Age**	34.3 ± 12.2	33.9 ± 11.1	<0.05	0.9	−0.1	0.9
	**Weight**	57.7 ± 14.2	63.1 ± 12.0	2.8	0.1	1.7	0.1
	**Height**	158.6 ± 6.9	158.7 ± 5.2	<0.05	0.9	0.1	0.9
	**BMI**	23.2 ± 6.4	25.2 ± 5.2	2.0	0.2	1.4	0.2
	**PhA**	5.7 ± 0.2	4.9 ± 0.5	73.7	<0.05	8.6	<0.05
	**R/H**	335.9 ± 41.1	329.2 ± 74.6	12.6	<0.05	−3.5	<0.05
	**Xc/H**	36.2 ± 8.5	34.6 ± 9.2	2.5	0.1	−1.6	0.1

**Table 3:** Analysis of mean (robust Welch’s test) and variance between healthy, non-severe and severe Dengue patients. (*p*-value less than 0.05).

	Study Groups	Helathy	Non-Severe	Severe	ANOVA Test		Student *t *Test	
					F	*p*-Value	T	*p*-Value
***Males***								
**Mean ± SD**	**Weight**	70.4 ± 9.9	64.9 ± 14.7	60.0 ± 14.9	4.6	<0.05	3.9	<0.05
	**ECF/ICF**	0.7 ± 0.1	0.8 ± .03	0.9 ± 0.1	0.1	121.2	<0.05	81.6
	**PhA**	7.1 ± 0.5	6.7 ± 0.3	5.2 ± 0.7	223.5	<0.05	151.6	<0.05
	**R/H**	289.9 ± 43.3	288.5 ± 57.4	258.1 ± 48.1	6.8	<0.05	6.8	<0.05
	**Xc/H**	34.2 ± 5.0	33.1 ± 6.7	31.9 ± 7.2	2.5	0.1	2.244	0.1
***Females***								
**Mean ± SD**	**Weight**	65.7 ± 12.5	63.1 ± 11.9	57.7 ± 14.2	1.9	0.2	1.6	0.2
	**ECF/ICF**	0.8 ± 0.03	0.9 ± 0.03	1.0 ± 0.08	235.8	<0.05	85.9	<0.05
	**PhA**	5.8 ± 0.5	5.6 ± 0.2	4.8 ± 0.5	58.9	<0.05	61.5	<0.05
	**R/H**	376.7 ± 62.4	335.9 ± 41.0	329.1 ± 74.6	9.5	<0.05	10.8	<0.05
	**Xc/H**	41.1 ± 6.6	36.2 ± 8.4	34.6 ± 9.2	0.7	0.5	0.5	0.6

## Abbreviations

The following abbreviations are used in this manuscript:

BIVA	Bioimpedance vector analysis
BMI	Body mass index
ECF	Extracellular fluid
ICF	Intracellular fluid
